# Supplementary material for: HIVprotI: an integrated web based platform for prediction and design of HIV proteins inhibitors
Source: J Cheminform. 2018 Mar 9;10:12. doi: 10.1186/s13321-018-0266-y (PMC5845081; doi:10.1186/s13321-018-0266-y)
Supplement: Supplementary file 2 — Additional file 2. Source code of HIVProtI web server. [file 13321_2018_266_MOESM2_ESM.zip › HIVprotI_Source-code/hivprotI/weka-3-6-6/doc/weka/classifiers/trees/j48/ClassifierTree.html]

ClassifierTree


---


|  |  |  |
| --- | --- | --- |
| **Overview** \| **Package** \| **Class** \| **Tree** \| **Deprecated** \| **Index** \| **Help** \| **Weka's home** | |  |
| **PREV CLASS**   **NEXT CLASS** | **FRAMES**    **NO FRAMES**     **All Classes** |  |
| SUMMARY: NESTED \| FIELD \| CONSTR \| METHOD | DETAIL: FIELD \| CONSTR \| METHOD |  |


---


## weka.classifiers.trees.j48 Class ClassifierTree

```
java.lang.Object
  weka.classifiers.trees.j48.ClassifierTree
```

**All Implemented Interfaces:**: java.io.Serializable, CapabilitiesHandler, Drawable, RevisionHandler

**Direct Known Subclasses:**: C45PruneableClassifierTree, C45PruneableClassifierTreeG, NBTreeClassifierTree, PruneableClassifierTree

---

``` public class ClassifierTree extends java.lang.Object implements Drawable, java.io.Serializable, CapabilitiesHandler, RevisionHandler ```

Class for handling a tree structure used for
classification.

**Version:**
:   $Revision: 5531 $

**Author:**
:   Eibe Frank (eibe@cs.waikato.ac.nz)

**See Also:**: Serialized Form

---

**Field Summary**

**Fields inherited from interface weka.core.Drawable**

`BayesNet, Newick, NOT_DRAWABLE, TREE`


**Constructor Summary**

`ClassifierTree(ModelSelection toSelectLocModel)`
  
          Constructor.


| **Method Summary** | |
| --- | --- |
| `int` | `assignIDs(int lastID)`             Assigns a uniqe id to every node in the tree. |
| `void` | `buildClassifier(Instances data)`             Method for building a classifier tree. |
| `void` | `buildTree(Instances data, boolean keepData)`             Builds the tree structure. |
| `void` | `buildTree(Instances train, Instances test, boolean keepData)`             Builds the tree structure with hold out set |
| `double` | `classifyInstance(Instance instance)`             Classifies an instance. |
| `void` | `cleanup(Instances justHeaderInfo)`             Cleanup in order to save memory. |
| `double[]` | `distributionForInstance(Instance instance, boolean useLaplace)`             Returns class probabilities for a weighted instance. |
| `Capabilities` | `getCapabilities()`             Returns default capabilities of the classifier tree. |
| `java.lang.String` | `getRevision()`             Returns the revision string. |
| `java.lang.String` | `graph()`             Returns graph describing the tree. |
| `int` | `graphType()`             Returns the type of graph this classifier represents. |
| `int` | `numLeaves()`             Returns number of leaves in tree structure. |
| `int` | `numNodes()`             Returns number of nodes in tree structure. |
| `java.lang.String` | `prefix()`             Returns tree in prefix order. |
| `java.lang.StringBuffer[]` | `toSource(java.lang.String className)`             Returns source code for the tree as an if-then statement. |
| `java.lang.String` | `toString()`             Prints tree structure. |

**Methods inherited from class java.lang.Object**

`equals, getClass, hashCode, notify, notifyAll, wait, wait, wait`

**Constructor Detail**

### ClassifierTree

```
public ClassifierTree(ModelSelection toSelectLocModel)
```

:   Constructor.


**Method Detail**

### getCapabilities

```
public Capabilities getCapabilities()
```

:   Returns default capabilities of the classifier tree.

    :   **Specified by:**: `getCapabilities` in interface `CapabilitiesHandler`
    :   **Returns:**: the capabilities of this classifier tree **See Also:**: `Capabilities`

---


### buildClassifier

```
public void buildClassifier(Instances data)
                     throws java.lang.Exception
```

:   Method for building a classifier tree.

    :   **Parameters:**: `data` - the data to build the tree from **Throws:**: `java.lang.Exception` - if something goes wrong

---


### buildTree

```
public void buildTree(Instances data,
                      boolean keepData)
               throws java.lang.Exception
```

:   Builds the tree structure.

    :   **Parameters:**: `data` - the data for which the tree structure is to be generated.: `keepData` - is training data to be kept? **Throws:**: `java.lang.Exception` - if something goes wrong

---


### buildTree

```
public void buildTree(Instances train,
                      Instances test,
                      boolean keepData)
               throws java.lang.Exception
```

:   Builds the tree structure with hold out set

    :   **Parameters:**: `train` - the data for which the tree structure is to be generated.: `test` - the test data for potential pruning: `keepData` - is training Data to be kept? **Throws:**: `java.lang.Exception` - if something goes wrong

---


### classifyInstance

```
public double classifyInstance(Instance instance)
                        throws java.lang.Exception
```

:   Classifies an instance.

    :   **Parameters:**: `instance` - the instance to classify **Returns:**: the classification **Throws:**: `java.lang.Exception` - if something goes wrong

---


### cleanup

```
public final void cleanup(Instances justHeaderInfo)
```

:   Cleanup in order to save memory.

    :   **Parameters:**: `justHeaderInfo` -

---


### distributionForInstance

```
public final double[] distributionForInstance(Instance instance,
                                              boolean useLaplace)
                                       throws java.lang.Exception
```

:   Returns class probabilities for a weighted instance.

    :   **Parameters:**: `instance` - the instance to get the distribution for: `useLaplace` - whether to use laplace or not **Returns:**: the distribution **Throws:**: `java.lang.Exception` - if something goes wrong

---


### assignIDs

```
public int assignIDs(int lastID)
```

:   Assigns a uniqe id to every node in the tree.

    :   **Parameters:**: `lastID` - the last ID that was assign **Returns:**: the new current ID

---


### graphType

```
public int graphType()
```

:   Returns the type of graph this classifier
    represents.

    :   **Specified by:**: `graphType` in interface `Drawable`
    :   **Returns:**: Drawable.TREE

---


### graph

```
public java.lang.String graph()
                       throws java.lang.Exception
```

:   Returns graph describing the tree.

    :   **Specified by:**: `graph` in interface `Drawable`
    :   **Returns:**: the tree as graph **Throws:**: `java.lang.Exception` - if something goes wrong

---


### prefix

```
public java.lang.String prefix()
                        throws java.lang.Exception
```

:   Returns tree in prefix order.

    :   **Returns:**: the prefix order **Throws:**: `java.lang.Exception` - if something goes wrong

---


### toSource

```
public java.lang.StringBuffer[] toSource(java.lang.String className)
                                  throws java.lang.Exception
```

:   Returns source code for the tree as an if-then statement. The
    class is assigned to variable "p", and assumes the tested
    instance is named "i". The results are returned as two stringbuffers:
    a section of code for assignment of the class, and a section of
    code containing support code (eg: other support methods).

    :   **Parameters:**: `className` - the classname that this static classifier has **Returns:**: an array containing two stringbuffers, the first string containing assignment code, and the second containing source for support code. **Throws:**: `java.lang.Exception` - if something goes wrong

---


### numLeaves

```
public int numLeaves()
```

:   Returns number of leaves in tree structure.

    :   **Returns:**: the number of leaves

---


### numNodes

```
public int numNodes()
```

:   Returns number of nodes in tree structure.

    :   **Returns:**: the number of nodes

---


### toString

```
public java.lang.String toString()
```

:   Prints tree structure.

    :   **Overrides:**: `toString` in class `java.lang.Object`
    :   **Returns:**: the tree structure

---


### getRevision

```
public java.lang.String getRevision()
```

:   Returns the revision string.

    :   **Specified by:**: `getRevision` in interface `RevisionHandler`
    :   **Returns:**: the revision


---


|  |  |  |
| --- | --- | --- |
| **Overview** \| **Package** \| **Class** \| **Tree** \| **Deprecated** \| **Index** \| **Help** \| **Weka's home** | |  |
| **PREV CLASS**   **NEXT CLASS** | **FRAMES**    **NO FRAMES**     **All Classes** |  |
| SUMMARY: NESTED \| FIELD \| CONSTR \| METHOD | DETAIL: FIELD \| CONSTR \| METHOD |  |


---
